# Identification and characterization of a natural polymorphism in *FT-A2* associated with increased number of grains per spike in wheat

**DOI:** 10.1007/s00122-021-03992-y

**Published:** 2021-11-26

**Authors:** Priscilla Glenn, Junli Zhang, Gina Brown-Guedira, Noah DeWitt, Jason P. Cook, Kun Li, Eduard Akhunov, Jorge Dubcovsky

**Affiliations:** 1 Department of Plant Sciences, University of California, Davis, CA 95616 USA; 2 USDA-ARS Plant Science Research, Raleigh, NC 27695 USA; 3 Department of Crop and Soil Sciences, North Carolina State University, Raleigh, NC 27695 USA; 4 Department of Plant Sciences and Plant Pathology, Montana State University, Bozeman, MT USA; 5 Howard Hughes Medical Institute, Chevy Chase, MD 20815 USA; 6 Department of Plant Pathology, Kansas State University, Manhattan, KS 66506 USA

## Abstract

**Key message:**

We discovered a natural *FT-A2* allele that increases grain number per spike in both pasta and bread wheat with limited effect on heading time.

**Abstract:**

Increases in wheat grain yield are necessary to meet future global food demands. A previous study showed that loss-of-function mutations in *FLOWERING LOCUS T2* (*FT2*) increase spikelet number per spike (SNS), an important grain yield component. However, these mutations were also associated with reduced fertility, offsetting the beneficial effect of the increases in SNS on grain number. Here, we report a natural mutation resulting in an aspartic acid to alanine change at position 10 (D10A) associated with significant increases in SNS and no negative effects on fertility. Using a high-density genetic map, we delimited the SNS candidate region to a 5.2-Mb region on chromosome 3AS including 28 genes. Among them, only *FT-A2* showed a non-synonymous polymorphism (D10A) present in two different populations segregating for the SNS QTL on chromosome arm 3AS. These results, together with the known effect of the *ft-A2* mutations on SNS, suggest that variation in *FT-A2* is the most likely cause of the observed differences in SNS. We validated the positive effects of the A10 allele on SNS, grain number, and grain yield per spike in near-isogenic tetraploid wheat lines and in an hexaploid winter wheat population. The A10 allele is present at very low frequency in durum wheat and at much higher frequency in hexaploid wheat, particularly in winter and fall-planted spring varieties. These results suggest that the *FT-A2* A10 allele may be particularly useful for improving grain yield in durum wheat and fall-planted common wheat varieties.

**Supplementary Information:**

The online version contains supplementary material available at 10.1007/s00122-021-03992-y.

## Introduction

Wheat is a global crop of major economic value and nutritional importance as it provides around 20% of the calories and protein consumed by the human population (http://www.fao.org/faostat/en/#data/FBS). However, with ever-changing environmental conditions and the rising human population, it is critical to increase wheat grain yield to meet future demands. Yield is a multifaceted trait that can be partitioned into several yield components, including spikes per unit of area, spikelet number per spike (SNS), grains per spikelet, and grain weight. Several genes have been identified that affect these grain yield components (Kuzay et al. 2019; Li et al. 2019; Poursarebani et al. 2015; Sakuma et al. 2019; Shaw et al. 2013; Simmonds et al. 2016; Wang et al. 2019).

Many of the genes affecting SNS also have strong effects on flowering time that can limit their use in variety development. Flowering before or after the optimum flowering time can result in yield penalties due to reduced fertility or increased risks of frost or heat damage, respectively. The vernalization gene *VRN1* is a good example of a gene affecting both flowering time and SNS. The *vrn1*-null mutant significantly increases SNS by delaying the transition of the inflorescence meristem to a terminal spikelet, but also delays the transition of the vegetative meristem to inflorescence meristem, resulting in a very late heading time (Li et al. 2019). Another good example is the main wheat photoperiod gene *PHOTOPERIOD1* (*PPD1)*, which shows a strong correlation between heading date and SNS in lines carrying different dosages of *PPD1* loss-of-function mutations (*R*^*2*^ = 0.74) (Shaw et al. 2013). A correlation between heading date and SNS has also been observed in genes regulated by *PPD1* such as the *FLOWERING LOCUS T1* gene (*FT1*) (Brassac et al. 2021; Finnegan et al. 2018; Isham et al. 2021; Lv et al. 2014)*.*

*FT1* encodes a mobile protein that travels through the phloem and carries environmental signals from the leaves to the shoot apical meristem (SAM), where it forms a complex with 14–3-3 and FD-like proteins (Florigen Activation Complex) (Taoka et al. 2011). This complex binds to the promoter of the meristem identity gene *VERNALIZATION1* (*VRN1)*, promoting its expression and the transition from the vegetative to the reproductive phase in wheat (Li et al. 2015). Induction of *FT1* also results in the upregulation of *SUPPRESSOR OF OVEREXPRESSION OF CONSTANS1-1* (*SOC1*), *LEAFY* (LFY) and genes in the gibberellin (GA) pathway that are essential for spike development and stem elongation (Pearce et al. 2013). A deletion of *FT-B1* in hexaploid wheat delays the transition to reproductive growth and increases SNS (Finnegan et al. 2018).

In addition to *FT1*, wheat has at least five *FT-like* paralogs designated as *FT2* to *FT6* (Lv et al. 2014), which have some overlapping functions but also varying degrees of sub-functionalization (Halliwell et al. 2016; Lv et al. 2014). *FT2* is the most similar paralog to *FT1* (78% protein identity), but the two genes exhibit marked differences in transcription and protein interaction profiles. Whereas the FT1 protein interacts with five out of the six wheat 14-3-3 proteins tested so far, FT2 failed to interact with any of these members of the Florigen Activation Complex (Li et al. 2015). The two genes also differ in their temporal and spatial transcription profiles. *FT1* transcript levels in the leaves are upregulated earlier than *FT2* when plants are grown at room temperature, but only *FT2* is induced when plants are grown for a long period at 4 °C (vernalization) (Shaw et al. 2019). Interestingly, *FT2* is the only member of the wheat *FT-like* gene family that is expressed directly in the shoot apical meristem (SAM) and in the developing spike (Lv et al. 2014), in addition to leaves and elongating stems (Fig. S1).

Loss-of-function mutations in *FT2*, identified in a sequenced mutant population of the tetraploid wheat variety Kronos (Krasileva et al. 2017), resulted in limited differences in heading time but significantly increased SNS (Shaw et al. 2019). Similar increases in SNS were observed in *ft-B2* natural mutants detected in hexaploid wheat (Gauley et al. 2021). The loss-of function mutation in the A-genome copy of *FT2* (*FT-A2*) in Kronos was associated with significantly larger increases in SNS (10–15%) than the mutation in the B-genome copy (*FT-B2*, 2–5%). This difference in SNS was associated with much higher transcript levels of *FT-A2* relative to *FT-B2* in all tissues and developmental stages (Fig. S1). The increases in spikelet number in the double *ft-A2 ft-B2* mutant (henceforth *ft2*-null) were significantly larger than in the single *ft-A2* mutant confirming that the *FT-B2* gene still has a residual effect on SNS in spite of its lower transcript levels.

The increase in SNS in the *ft-A2* mutant was associated with reduced fertility, offsetting the potential positive effects of the increase in SNS on total grain yield (Shaw et al. 2019). This effect was observed in growth chambers under optimal conditions suggesting that is not an indirect effect of altered flowering time. We hypothesized that strong selection in cultivated wheat for grain yield might have selected an *FT-A2* variant with a positive effect on SNS, but without the associated negative effect on fertility. Analysis of natural variation in *FT-A2* revealed an aspartic acid to alanine change at position 10 (D10A) that was rare in tetraploid wheat but frequent in modern common wheat varieties, suggesting positive selection for the new allele. In this study, we characterized the effect of the D10A polymorphism on wheat heading time, SNS, grain number, and spike yield in different wheat classes and performed a high-density genetic mapping of the SNS QTL that identified *FT-A2* as the most likely candidate gene.

## Material and methods

Analysis of the exome capture data generated by the WheatCAP project using the assay developed by NimbleGen (Krasileva et al. 2017) and deposited in the Wheat T3 database (https://wheat.triticeaetoolbox.org/) revealed the existence of an A to C SNP within the *FT-A2* coding region that resulted in the D10A polymorphism. We studied the effect of this SNP on heading time, SNS, grain number, and spike yield in two segregating populations in tetraploid and hexaploid wheat.

### Biparental mapping population in tetraploid wheat (*Triticum turgidum* ssp. *durum*)

The tetraploid mapping population included 163 BC_1_F_2_ lines from the cross Kronos *2/Gredho (designated KxG hereafter). Kronos (PI 576168, *FT-A2* D10 allele) is a semi-dwarf (*Rht-B1b*), spring wheat with reduced photoperiod sensitivity (*Ppd-A1a*), whereas Gredho (PI 532239, *FT-A2* A10 allele) is a tall (*Rht-B1a*), photoperiod sensitive (*Ppd-A1b*) spring landrace from Oman. We planted the KxG population as headrows in the 2015–2016 season at the UC Experimental Field Station in Davis, CA, with each row including on average five individual plants.

### Near-isogenic lines of the *FT-A2* A10 allele from Gredho into Kronos

We also evaluated the effect of the *FT-A2* alleles in two sets of near-isogenic lines (NILs). For the first set, we selected *FT-A2* heterozygous BC_1_F_2_ and BC_1_F_3_ lines from the cross Kronos *2/Gredho and selected two sets of homozygous BC_1_F_3-4_ homozygous A10 and D10 sister lines using the *FT-A2* marker (H2-14 and H2-23). These lines were semi-dwarf and carried the *Ppd-A1a* allele for reduced photoperiod sensitivity and the *Vrn-A1* allele for spring growth habit. We used the BC_1_F_3-5_ grains produced by these plants for two field experiments, one at the University of California, Davis (UCD) and the other one at Tulelake (California northern intermountain region). Both field experiments were organized in a complete randomized design with plants as experimental units. Three to five spikes were measured per plant and averaged for 10 plants per genotype at the UC Davis experiment. In the Tulelake experiment, 23–27 spikes per genotype were randomly collected and used as experimental units in the statistical analyses.

In parallel, we backcrossed the A10 allele into Kronos for three additional generations (Kronos *5/Gredho) and then selected BC_4_F_2_ NILs homozygous for the A10 and D10 alleles using the *FT-A2* molecular marker. The BC_4_F_3_ seed was increased in the greenhouse in 2020, and the BC_4_F_4_ grains were used for field experiments at UCD and Tulelake in 2021. These experiments used small plots (four 1-m rows, 1.1 m^2^) as experimental units, organized in a randomized complete block design with 12 blocks. Grains of the BC_4_F_4_ Kronos isogenic line with the A10 allele were deposited in the National Small Grain Collection as PI 699107.

### Biparental mapping population in hexaploid winter wheat

The hexaploid population included 358 F_5_-derived recombinant inbred lines (RILs) from the cross between soft-red winter wheat lines LA95135 (CL-850643/PIONEER-2548//COKER9877/3/FL-302/COKER-762) x SS-MVP57 (FFR555W/3/VA89-22-52/TYLER//REDCOAT*2/GAINES). LA95135 is semidwarf (*Rht-D1b*) and photoperiod sensitive (*Ppd-D1b*), whereas SS-MVP57 is tall (*Rht-D1a*) and has reduced photoperiod sensitivity (*Ppd-D1a*) (DeWitt et al. 2021). This winter wheat population was previously genotyped and phenotyped as 1-m rows in the field at Raleigh, NC, and Kinston, NC, during the 2017–2018 season, and in Raleigh, Kinston, and Plains, GA, in the 2018–2019 season (DeWitt et al. 2021). These locations will be referred to as Raleigh (Ral), Kinston (Kin), and Plains (Pla) followed by the harvest year (18 or 19).

### *FT-A2* marker development and allelic frequencies

We targeted the *FT-A2*, D10A SNP at position 124,172,909 bp (RefSeq v1.0) on chromosome 3A with a Cleaved Amplified Polymorphic Sequence (CAPS) marker. Primers FT-A2-D10A forward and reverse (Table S1) amplify a fragment of 705 bp. After digestion with the restriction enzyme *Apa*I, the fragment amplified from the D10 allele remained undigested, whereas the fragment amplified from the A10 allele was digested into two fragments of 448 and 257 bp.

We used this marker to determine the frequency of the D10A mutation in 89 *T. urartu*, 82 *T. turgidum* ssp. *dicoccoides,* 32 *T. turgidum* ssp. *dicoccon,* 417 *T. turgidum* ssp. *durum,* and 705 *T. aestivum* accessions summarized in Supplementary Appendix S1. Among the hexaploid lines, we included a collection of 238 landraces and varieties (He et al. 2019) and a set of 126 winter wheats (T3/Wheat) genotyped by exome capture and with data for the *FT-A2* D10A polymorphism. We also used the *FT-A2* marker to genotype a panel of 242 spring wheats with reduced photoperiod sensitivity (Zhang et al. 2018) and a panel of 99 varieties and modern breeding lines from the Montana State University wheat breeding program (Supplementary Appendix S1). Based on the planting season used in the area where the spring varieties were developed, they were divided into those developed under spring planting (hereafter "DuS") or under fall planting (hereafter “DuF”). A previous study has shown that DuS and DuF groups are genetically differentiated using the 90 K SNP array (Zhang et al. 2018) (Supplementary Appendix S1).

### High resolution genetic map

The high-resolution map of the KxG population was developed in two phases. In the first phase, we identified two BC_1_F_3_ plants from the KxG BC_1_F_2_ head rows, H2 and D12, which were heterozygous for *FT-A2* candidate region. From these heterozygous lines, we generated large segregating Heterogeneous Inbred Families (HIF) populations to identify recombination events within the *FT-A2* candidate region. For phenotypic screenings, these recombinants were space-planted at least three inches apart in a completely randomized design. Additional markers in the candidate gene region were developed for 11 genes on both sides of *FT-A2* covering a region of ~ 10 Mb using the exome capture sequence data from Kronos and Gredho (Table S1).

### Statistical analysis

In the tetraploid biparental population, we analyzed the effect of the *FT-A2* alleles using a 3 × 2 factorial ANOVA. This analysis included the genotypic variation at *PPD-A1* and *RHT-B1* as additional factors, since both genes are known to have pleiotropic effects on heading time and yield components. In the hexaploid winter wheat population, we analyzed the effect of *FT-A2* in a 4 × 2 factorial ANOVA including the segregating genes *PPD-D1*, *RHT-D1,* and *WHEAT ORTHOLOG OF APO1* (*WAPO-A1*), which was previously shown to affect SNS (Kuzay et al. 2019). Analysis of Variance was conducted with the “Anova” function in R package “car” (Fox et al. 2019) with type 3 sum of squares.

### Yeast two-hybrid assays

Modified Gateway (Invitrogen) bait/prey vectors pLAW10 and pLAW11 (Cantu et al. 2013) and yeast strain Y2HGold (Clontech, Mountain View, CA, USA) were used in the yeast two-hybrid assays. pLAW10 is the Gateway version of pGBKT7 (GAL4 DNA-binding domain, BD), and pLAW11 is the Gateway version of pGADT7 (GAL4 activation domain, AD). For all Gateway compatible cloning, pDONR/Zeo (Life Technologies, Grand Island, NY, USA) was used to generate the entry vectors. All constructs were verified by sequencing. Yeast two-hybrid assays were performed according to the manufacturer’s instructions (Clontech). Transformants were selected on SD medium lacking leucine (− L) and tryptophan (− W) plates and re-plated on SD medium lacking –L, –W, histidine (− H), and adenine (− A) to test the interactions.

## Results

### Natural variation in *FT-A2*

We used exome capture data deposited in the T3/Wheat database (https://triticeaetoolbox.org/wheat/) to explore the natural polymorphisms in *FT-A2*. We identified an A to C SNP at position 124,172,909 in chromosome arm 3AS of the Chinese Spring (CS) RefSeq v1.0, which resulted in an amino acid change at position 10 of the FT-A2 protein from aspartic acid (D) to alanine (A) (henceforth, D10 and A10 alleles). In the analyzed accessions of *T. urartu*, *T. turgidum* ssp. *dicoccoides,* and *T. turgidum* ssp. *dicoccon*, we detected only the D10 allele (Table 1). D10 was also the only allele detected in all the other grass species we analyzed including *Lolium perenne* (AMB21802), *Oryza sativa* (XP_021310907), *Zea mays* (NP_001106251), and *Panicum virgatum* (APP89655), indicating that D10 is the ancestral allele. In this study, we describe the change from the ancestral to the derived allele (D10A) rather than relative to the Chinese Spring (CS) reference genome that carries the derived A10 allele,

**Table 1:** Frequency of the *FT-A2* alleles in different germplasm collections

Species	Ploidy	No. acc	A10%	D10%	A10	D10
*T. urartu*	2x	89	0.0%	100.0%	0	89
*T. turgidum* ssp. *dicoccoides*	4x	82	0.0%	100.0%	0	82
*T. turgidum* ssp. *dicoccon*	4x	32	0.0%	100.0%	0	32
*T. turgidum* ssp. *durum*	4x	417	0.7%	99.3%	3	414
*T. aestivum* Exome capture^a^	6x	238	59.7%	40.3%	142	96
*T. aestivum* US winter wheats^b^	6x	126	81.7%	18.3%	103	23
*T. aestivum* Spring DUF^c^	6x	149	58.4%	41.6%	87	62
*T. aestivum* Spring DUS^d^	6x	192	34.4%	65.6%	66	126

^a^He et al. (2019)

^b^T3/wheat

^c^Zhang et al. (2018)

^d^ Zhang et al. (2018) + 99 breeding lines from MT

We also screened a collection of 417 *T. turgidum* ssp. *durum* accessions with a CAPS marker for the D10A polymorphism (see “Material and methods”) and found that only 0.7% carried the A10 allele (Table 1). Two of the three accessions with the A10 allele were from Oman (PI 532239 = ‘Gredho’ and PI 532242, ‘Musane and Byaza’) and the other one was from Turkey (PI 167718), suggesting that the A10 allele is almost absent from modern Western durum germplasm.

We detected a higher frequency of the A10 allele (56.5%) among 705 *T. aestivum* ssp. *aestivum* lines (Table 1). This overall frequency was similar to that detected in a worldwide collection of landraces and varieties combining winter and spring lines (59.7%) (He et al. 2019). We also analyzed the frequency of the D10A polymorphisms in two collections with known growth habit and found a higher frequency of the A10 allele among the winter lines (81.7%) than among the spring lines (44.9%, Table 1). Among the 341 spring wheat lines genotyped with the *FT-A2* marker, we found that varieties developed under fall-planting (DuF or long cycle) had a significantly higher frequency of the A10 allele (58.4%) than those developed under spring-planting (DuS or short cycle, 34.4%, *χ*^2^ *P* < 0.001, Table 1). A complete list of the accessions used in these calculations is available in Supplementary Appendix 1, and a summary of the frequencies is presented in Table 1.

### Effect of the D10A polymorphism in tetraploid wheat

To test the effect of the D10A polymorphism on SNS, we used the diagnostic CAPS marker to screen 163 BC_1_F_2_ plants from the KxG population segregating for this polymorphism. We also genotyped this population with markers for the segregating *RHT-B1* (Guedira et al. 2010) and *PPD-A1* (Wilhelm et al. 2009) genes, which can also affect SNS. Plants were grown in the field in the 2015–2016 season in Davis, CA, and were phenotyped for individual plant height (HT), days to heading (DTH), and spikelet number per spike (SNS, Table 2).

**Table 2:** Effects of *FT-A2*, *PPD-A1* and *RHT-B1* on plant height (HT), days to heading (DTH), and spikelet number per spike (SNS)

		Plant height (HT, cm)	Days to heading (DTH)	Spikelet no./spike (SNS)
*FT-A2* LSmean ± s.e.m	Kronos (D10)	113.4 ± 3.2	130.5 ± 0.9	25.1 ± 0.5
	Gredho (A10)	118.3 ± 2.2	130.6 ± 0.6	26.7 ± 0.3
Three-way ANOVA	*P *value	ns	ns	*
*PPD-A1* LSmean ± s.e.m	Kronos	108.1 ± 2.3	120.8 ± 0.6	22.6 ± 0.4
	Gredho	121.6 ± 2.6	141.0 ± 0.7	29.6 ± 0.4
Three-way ANOVA	*P *value	***	***	***
*Rht-B1*	Kronos	97.1 ± 2.5	131.5 ± 0.7	26.2 ± 0.5
LSmean ± s.e.m	Gredho	131.4 ± 2.8	130.2 ± 0.8	25.8 ± 0.4
Three-way ANOVA	*P *value	***	*	ns

Three-way ANOVA with *P* values of the main effects and least-square means (LSmeans)

*ns*  not significant, *P < 0.05, **P < 0.01, ***P < 0.001

All the interactions were non-significant

The three-way factorial ANOVAs including *FT-A2, RHT-B1,* and *PPD-A1* as factors showed significant effects for SNS, HT, and DTH and no significant interactions for any of the traits. As expected, *RHT-B1* showed the strongest effect on plant height and *PPD-A1* on heading time, although both genes affected both traits (Table 2). The strongest effect on SNS was detected for *PPD-A1*, but a significant effect was also detected for *FT-A2* (Table 2), with plants homozygous for A10 showing 6.4% higher SNS than those homozygous for D10 allele (Table 2). The differences in SNS between the *FT-A2* alleles were larger in the late flowering plants homozygous for the photoperiod sensitive allele from Gredho (2.3 spikelets/spike) than in the early flowering plants homozygous for the Kronos allele for reduced photoperiod sensitivity (1.0 spikelets per spike), but the interaction was not significant.

### Effect of the *FT-A2* alleles in Kronos near-isogenic lines

To analyze the effect of the D10A polymorphism independently of the variability generated by other major genes, we evaluated two sets of near-isogenic lines in field experiments in 2020 (BC_1_F_3-5_ sister lines) and 2021 (BC_4_F_2:4_ sister lines, see “Material and methods”) at UCD and Tulelake. In the 2020 experiment at UCD, lines with the A10 allele showed large and significant increases in SNS (13.8%), grain number per spike (GNS, 31.7%), grains per spikelet (16.1%, also referred to as fertility), and grain yield per spike (33.0%) relative to the sister lines homozygous for the D10 allele (Table 3). The results from this experiment were consistent between two independent pairs of BC_1_F_3-5_ sister lines (H2-14 and H2-23, Table 3). The 2020 experiment in Tulelake (Northern California, spring planting) using BC_1_F_3-5_ sister lines from family H2-14 also showed a significant increase in SNS (4.0%), but the increases in GNS, grains per spikelet, and grain yield per spike were not significant (Table 3).

**Table 3:** Comparisons of near-isogenic lines with the *FT-A2* A10 and D10 alleles in field experiments at UC Davis and Tulelake in 2020 and 2021. All % changes are relative to D10

	Allele	*N*	SNS	GN	Grains/spikelet	GW mg	Yield/spike g	Yield/plot g
Davis 2020								
H2-14	D10	10^a^ (54 spikes)	20.27	59.22	2.92	55.81	3.31	NA
H2-14	A10	10^a^ (38 spikes)	21.92	70.77	3.23	56.78	4.07	NA
		A10% change	8.1**	19.6***	10.6**	1.8 ns	22.9**	
H2-23	D10	10^a^ (39 spikes)	19.36	56.31	2.89	54.47	3.07	NA
H2-23	A10	10^a^ (38 spikes)	23.11	81.06	3.51	54.21	4.41	NA
		A10% change	19.4***	44.0***	21.5***	−0.6 ns	43.6***	
Tulelake 2020								
H2-14	D10	27 spikes	17.15	44.11	2.57	38.26	1.69	NA
H2-14	A10	23 spikes	17.83	46.48	2.61	40.49	1.88	NA
		A10% change	4.0***	5.4 ns	1.4 ns	5.8 ns	11.2 ns	
Davis 2021								
BC_4_F_2:4_	D10	12^b^ (96 spikes)	18.52	67.85	3.67	60.34	4.09	1254
BC_4_F_2:4_	A10	12^b^ (96 spikes)	19.58	72.15	3.69	55.61	4.01	1251
		A10% change	5.7 **	6.3 *	0.5 ns	−7.8 ***	−2.0 ns	−0.2 ns
Tulelake 2021								
BC_4_F_2:4_	D10	12^b^ (96 spikes)	15.39	41.48	2.69	58.34	2.43	746
BC_4_F_2:4_	A10	12^b^ (96 spikes)	16.02	44.02	2.75	59.45	2.62	854
	Null	12^b^ (96 spikes)	16.54	40.39	2.44	54.07	2.20	789
		A10% change	4.1**	6.1**	2.1 ns	1.9 ns	7.8**	14.5 ns
		*ft2-*null % change	7.5***	–2.6 ns	−9.4***	−7.3***	−9.5**	5.8 ns

^a^Experimental units were 1 m rows, with 3–5 spikes measured per row

^b^Experimental units were 4 row plots (1.1 m^2^), with 8 spikes measured per plot

For the 2021 UCD experiment using 1.1-m^2^ small plots as experimental units (12 replications), BC_4_F_2:4_ lines with the A10 allele headed on average 0.8 d later than the sister lines with the D10 allele (*P* = 0.0252) and showed significant increases in SNS (5.7%, *P* = 0.0011) and GNS (6.3%, *P* = 0.0168, Table 3). In this experiment, we did not detect significant differences in grains per spikelet (*P* = 0.7919). We observed a negative correlation between average GNS and grain weight across the 24 plots (*R* = − 0.61) and a significant negative effect of the A10 allele on kernel weight (– 7.8%, *P* = 0.0002). The negative effect on grain weight offset the positive effect of the A10 allele on grain number resulting in non-significant differences in grain weight per spike or per plot (Table 3).

For the 2021 Tulelake experiment using 1.1-m^2^ small plots (12 replications), we included the Kronos lines with truncation mutations in *FT-A2* and *FT-B2* (*ft2-*null, henceforth) developed before (Shaw et al. 2019) in addition to the BC_4_F_2:4_ Kronos lines with the D10 and A10 alleles. The lines with the A10 allele showed highly significant increases in SNS (4.1%), GNS (6.1%), and grain yield per spike (7.7%) that were of similar magnitude to the ones observed in the 2020 Tulelake experiment (Table 3). The null line also showed a significant increase in SNS (7.5%) relative to the wildtype Kronos (D10), but the negative impact of the *FT2* loss-of-function mutations in grains per spikelet (−9.4%) and grain weight (−7.3%) resulted in a significant reduction in grain yield per spike (−9.3%, Table 3). No significant differences in grain yield per plot were detected among the three lines.

### The A10 allele has a positive effect on SNS and spike yield in winter wheat

To analyze the effect of the D10A *FT-A2* alleles in winter wheat, we used phenotypic data available from 358 F_5_-derived RILs from the cross between soft-red winter wheat lines LA95135 and SS-MVP57 (DeWitt et al. 2021) and genotypic data for the *FT-A2* marker developed in this study. This population was also segregating for *PPD-D1, RHT-D1*, and *WAPO-A1,* which were included as factors together with *FT-A2* in a 4 × 2 factorial ANOVA.

**Fig. 1:**
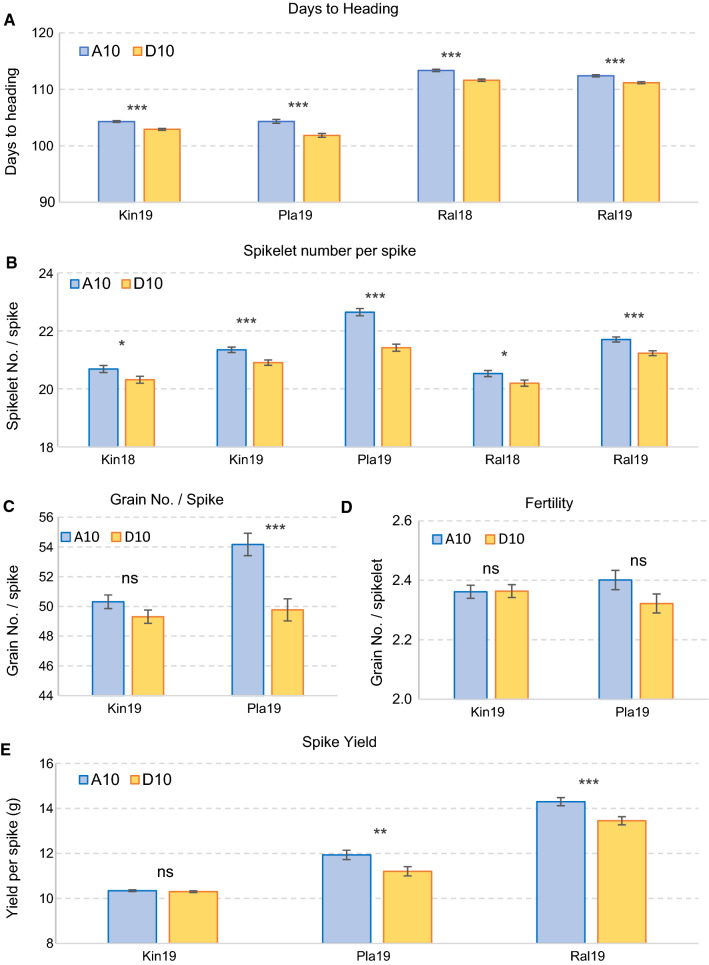
Comparison between *FT-A2* A10 (SS-MVP57) and D10 (LA95135) alleles in winter wheat. **a** Days to heading. **b** Spikelet number per spike. **c** Grain number per spike. **d** Grain number per spikelet (fertility). **e** Average spike yield. Bars are least square means from a factorial ANOVA including *PPD-*D1, *RHT-D1* and *WAPO-A1* as factors. Error bars are s.e.m. *ns* not significant, *P = 0.05, **P = 0.01, ***P = 0.001

Plants carrying the *FT-A2* allele A10 (SS-MVP57) headed on average 1.7 days later (*P* < 0.001, Fig. 1a) and had 0.6 more spikelet per spike (5.1% increase, *P* < 0.001, Fig. 1b) than plants carrying the D10 allele (LA95135). The differences in SNS were significant in all tested locations. The A10 allele was also associated with a significant increase in GNS in the overall ANOVA (*P* < 0.001), but the separate analyses of the two locations showed significant differences only at Pla19 (4.4 more grains per spike, *P* < 0.001, Fig. 1c). No significant effects were detected on fertility (Fig. 1d). A significant increase in spike yield was associated with the A10 allele in the overall ANOVA (average 4.6%, *P* < 0.001), and two out of the three tested locations were significant in the analyses by location (*P* < 0.001, Fig. 1e).

### High resolution mapping of the SNS QTL on chromosome 3AS

The previous results showed that the haplotypes associated with the *FT-A2* D10 and A10 alleles have a significant effect on SNS. To narrow down the candidate gene region and explore the linkage between the *FT-A2* D10A polymorphism and the differences in SNS, we generated a high-density map of the 3AS chromosome region in tetraploid wheat using a total of 3,161 BC_1_F_3_, BC_1_F_4_, and BC_1_F_5_ plants derived from the KxG population. These plants were screened in separate batches over three years using flanking markers 3A-117.83 and 3A-127.82 (numbers indicate coordinates in RefSeq v1.0 in Mb). Within this 9.9-Mb region including *FT-A2* (124.17 Mb), we identified 76 recombination events corresponding to a genetic distance of 1.58 cM (6.26 Mb per cM). One of these recombination events (H2-6-#14-5) was detected in the progeny test of primary recombinant H2-#6, which explains the presence of two close recombination events in this line (Table 4).

**Table 4:**
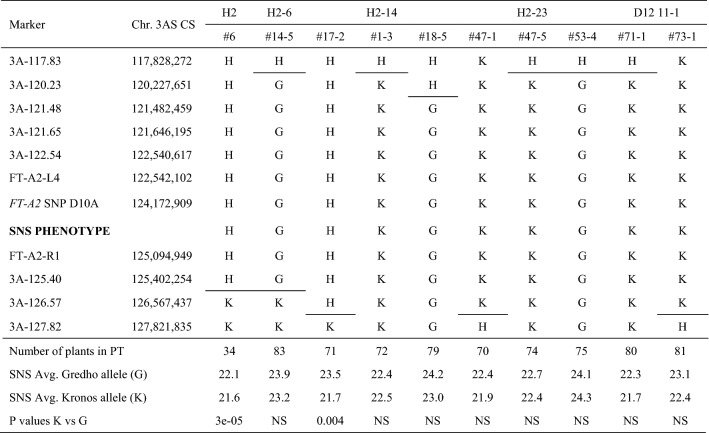
Critical recombinant BC_1_F_5_ from Davis 2019–2020 field seasons

All lines except recombinant H2 #6 were evaluated in the 2020 field season. Comparisons of SNS for statistical significance are only between sister lines segregating for the heterozygous region

In addition to the molecular marker for the *FT-A2* D10A SNP and the two flanking markers, we developed eight more KASP and CAPS markers in the candidate region (Table S1) and used them to genotype plants carrying recombination events in the region. The lines with the 10 closest recombination events to *FT-A2* are presented in Table 4 together with the results of the field progeny tests for SNS. Progenies of the lines H2-#6 and H2-14#17-2 heterozygous for *FT-A2* showed significant differences in SNS (*P* < 0.01) between lines homozygous for the two parental alleles, whereas progeny tests for the eight lines homozygous for *FT-A2* did not show significant difference in SNS between parental alleles in the heterozygous flanking regions (Table 4). Average SNS were as expected, with the lines homozygous for the A10 allele having 1.3 more spikelets on average than the lines homozygous for the D10 allele.

The phenotype of the critical recombinant line #18-5 with the closest distal recombination event to *FT-A2* was validated in a separate experiment in Davis in 2021 (Table S2). In this experiment, control lines showed highly significant differences in SNS (*P* < 0.0001) confirming that the differences in SNS were detectable in this experiment. By contrast, there was no significant difference between the sister lines with and without the recombination event #18-5, with both lines showing SNS values similar to the control line with the Gredho allele (Table S2). Taken together, these results confirmed that the causal gene for the 3AS QTL for SNS was proximal to the marker located at CS RefSeq v1.0 coordinate 120,227,651 (Table 4).

Later, we identified an additional line (BC_1_F_4_ H2-18 #28-4) with a closer recombination event to *FT-A2* in the proximal region between *FT-A2-R1* and *3A-125.4*, which we planted in a separate field experiment at Tulelake in the spring of 2020. This experiment included homozygous sister lines #28-4-1 and #28-4-3 fixed for either the Kronos or Gredho alleles in the segregating proximal region (Table 5) and as controls sister lines derived from plant #17-2 (Table 4) that were either homozygous for the *FT-A2* D10 (#17-2-18) or A10 allele (#17-2-22, Table 5). These two lines showed highly significant differences in SNS (*P* < 0.0001, Table 5) confirming that it was possible to detect differences between the two *FT-A2* alleles in this experiment. By contrast, there was no significant difference between the H2-18 #28-4 recombinant sister lines, confirming that the candidate gene was still linked to *FT-A2* (Table 5)*.* Based on this result, we established a closer proximal flanking marker (3A-125.4) and reduced the candidate region for the 3AS QTL to a 5.2 Mb interval between coordinates 120,227,651 and 125,402,254 (Table 5).

**Table 5:**
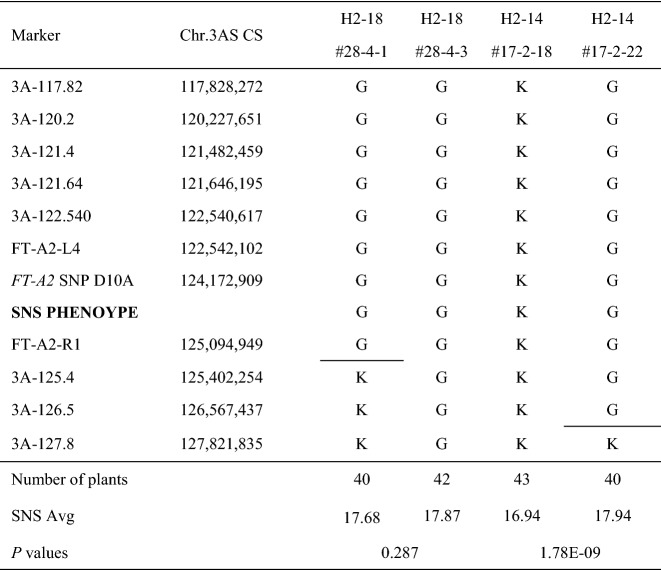
Spikelet number per spike (SNS) evaluation of BC_1_F_6_ homozygous sister lines from recombinant line H2-18 #28-4 in Tulelake 2020

Sister line #28-4-#1 carried a proximal Kronos chromosome segment and sister line #28-4-#3 a proximal Gredho chromosome segment. Lines #17-2-18 (*FT-A2* D10) and #17-2-22 (*FT-A2* A10) were included as controls

### Genes in the candidate gene region for the 3AS QTL for SNS

The annotated Chinese Spring reference genome region (RefSeq v1.1) between the two flanking markers defined in the previous section encompasses 28 high-confidence genes (including flanking genes *TraesCS3A02G141000* and *TraesCS3A02G143700*). The exome capture data revealed non-synonymous SNPs between Kronos and Gredho in only three out of the 28 genes, including the D10A polymorphism in *FT-A2.* The other two genes are described briefly below.

*TraesCS3A02G142200* encodes a leucine-rich repeat receptor-like protein kinase, so it is difficult to predict its potential effects. The predicted R872H amino acid change in Kronos (RefSeq v1.1 3AS 121,646,195) is in a conserved region close to the end of the protein (893 amino acids) and has a BLOSUM62 score of 0, predictive of a low probability of changes in protein structure or function. The R872H polymorphism was not detected in the parental lines LA95135 and SS-MVP57 of the hexaploid winter wheat populations segregating for the 3AS SNS QTL.

*TraesCS3A02G143600* encodes a short peptide (104 amino acids) with a polymorphism in Kronos that generates a premature stop codon (S59*, RefSeq v1.1 3AS 125,094,949 C to A). However, the predicted protein in Gredho also seems to be truncated since it is much shorter (104 amino acids) than the orthologous protein in wild emmer (XP_037404892.1, 483 amino acids) or *T. urartu* (EMS53367.1, 348 amino acids). In addition, the 104 amino acids in Gredho showed no similarity to other plant proteins in the GenBank nr database in species outside the genus *Triticum*, suggesting that *TraesCS3A02G143600* encodes a non-functional protein in both Kronos and Gredho. Similar to R872H, the S59* polymorphism was not detected in winter lines LA95135 and SS-MVP57.

The QTLs in the KxG and LA95135 x SS-MVP57 segregating populations are co-located and affect the same traits, so we hypothesized that they should have mutations in the same gene. Therefore, we prioritized genes carrying mutations in both populations. The predicted R872H amino acid change in *TraesCS3A02G142200* was polymorphic in the KxG population but not in the winter wheat population, and the same was true for the S59* premature stop codon in *TraesCS3A02G143600.* By contrast, the D10A polymorphism in FT-A2 (*TraesCS3A02G143100*) was detected in both mapping populations.

The R872H and S59* polymorphisms were found in tetraploid wheat but were not detected in any of the sequenced hexaploid wheats in the wheat PanGenome project (Walkowiak et al. 2020). By contrast, *FT-A2* was polymorphic in the same group of varieties, with CDC Landmark, Lancer, and Spelt carrying the D10 allele and CS, Julius, Jagger, CDC Stanley, ArinaLRFor, Mace, Norin 61, and SY Mattis carrying the *FT-A2* A10 allele. The previous observations, together with the known effect of *FT2* mutations on SNS (Shaw et al. 2019), suggest that *FT-A2* is the most likely candidate gene in this region. However, we cannot rule out the possibility of polymorphisms shared between the two populations in the regulatory regions of other candidate genes.

### Effect of the D10A polymorphism on FT-A2 interactions with 14-3-3 proteins

Previous results have shown positive interactions between FT1 and six of the seven 14-3-3 proteins tested, whereas FT-A2 did not interact with any of the 14-3-3 proteins (Li et al. 2015). This was a puzzling result because all other four FT-like genes showed positive interactions with at least one 14-3-3 protein. Since the original study was done using only the FT-A2 D10 allele, we decided to explore the effect of the A10 allele. In this study, both the FT-A2-D10 and FT-A2-A10 proteins failed to interact with any of the six tested 14-3-3 proteins, whereas the FT1 positive control showed a strong interaction signal (Fig. S2). No autoactivation was observed in the negative controls. Given the lack of interactions between both FT-A2 alleles and any of the tested 14-3-3 protein, we have initiated Y2H screens to test if there are other protein partners of FT-A2.

## Discussion

### Candidate gene and causal polymorphism

In this study, we focused on SNS for the high-density mapping because this trait has a higher heritability (*h* > 0.8) than other yield components (Kuzay et al. 2019; Zhang et al. 2018). Spikelet number per spike is determined early after the transition from the vegetative to the reproductive phase, when the spike meristem transitions into a terminal spikelet (Li et al. 2019). This limits the influence of later environmental variability on SNS relative to GNS or grain weight, which are affected by fertility, grain abortions, and conditions affecting grain filling until the end of the season.

The high heritability of SNS helped us to Mendelize the QTL (using large progeny tests) and to generate a high-density map of the SNS QTL on chromosome arm 3AS. We established a 5.2 Mb candidate gene region on chromosome arm 3AS including 28 annotated high-confidence genes in CS, including three with non-synonymous polymorphisms between Kronos (D10) and Gredho (A10): *TraesCS3A02G143100* (D10A), *TraesCS3A02G142200* (R872H), and *TraesCS3A02G143600* (S59*). The last two polymorphism were detected in the Kronos x Gredho population but not in the LA95135 (D10) and SS-MVP57 (A10) suggesting that they are unlikely candidate genes for the SNS QTL. In additions, the S59* and R872H polymorphisms were not detected among the varieties sequenced in the wheat pangenome (Walkowiak et al. 2020), which suggests that they originated in durum wheat and that the A10 mutation occurred in a haplotype different from the one present in modern durum wheat varieties (S59*-R872H haplotype).

After the elimination of these two genes, *FT-A2* is the only gene in the candidate region that has a non-synonymous polymorphism (D10A) linked to the differences in SNS in both mapping populations. Although we cannot rule out the possibility of polymorphisms in regulatory regions of other candidate genes affecting SNS in both populations, the genetic data presented here, together with the known effect of the loss-of-function mutations in *FT-A2* on SNS (Shaw et al. 2019), point to *FT-A2* as the most likely candidate gene for the SNS QTL.

The D10A amino acid change in FT-A2 has a BLOSUM 62 score of -2 and is located in a conserved region of the protein, suggesting a high probability of an effect on either protein structure or function. To test if any other polymorphisms in *FT-A2* were associated with the D10A polymorphism, we compared the available exons, introns, 5’ upstream region (5000 bp) and 3’ downstream region (2000 bp) of *FT-A2* in genomic sequences of hexaploid wheat (Walkowiak et al. 2020). We did not find any additional SNPs that differentiate the varieties with the D10 allele (CDC Landmark, Lancer and Spelta) from those carrying the A10 allele (CS, Julius, Jagger, CDC Stanley, ArinaLRFor, Mace, Norin 61, and SY Mattis) in the analyzed regions. Although we cannot completely rule out the possibility of polymorphisms located in regulatory regions outside the investigated region, the available evidence points to D10A as the most likely causal polymorphism. A conclusive test of this hypothesis will require the editing of the A124,172,909C, but this is not simple because this is a transversion, and currently available plant gene editors are not efficient to edit transversions. New prime editing technologies (Anzalone et al. 2019) may solve this problem once they become more efficient in plants (Lin et al. 2020).

### Differential recombination rates within the candidate gene region

The distribution of recombination events (RE) in the 10-Mb region between the flanking markers used in this study was not uniform. In the 2.4 Mb distal to the candidate gene region (117.8 to 120.2 Mb, 14 genes), we detected 56 RE resulting in an average of 23.3 RE/Mb or 4.0 RE/gene. In the 2.4 Mb proximal to the candidate region (125.4 to 127.8 Mb, 13 genes), we detected 20 RE resulting in a frequency of 8.3 RE /Mb or 1.5 RE/gene. Surprisingly, not a single RE was detected in the 5.2-Mb central candidate region (120.2 to 125.4 Mb, 28 genes), despite being twice as large and including twice the number of genes as the flanking regions. Recombination events occur mainly in gene regions (Darrier et al. 2017), so we would have expected to find 39 of the 76 RE within the candidate region if RE were distributed proportionally to the number of genes. The same number would be expected if RE were distributed proportionally to the physical length of the interval.

To explore if this lack of recombination in the central region was caused by a structural rearrangement, we used the sequenced genome of the tetraploid variety Svevo (Maccaferri et al. 2019) that showed the same SNPs as the Kronos exome capture across the candidate gene region. Since Gredho showed very few polymorphisms with CS across the candidate gene region, we compared the genomes of CS (A10) and Svevo (D10) in this region. In Svevo, we found orthologs to the 28 high confidence genes present in CS, with the exception of *TraesCS3A02G142500* that was present in the correct position and strand in Svevo (100% identical over all its length) but was not annotated. All the genes were in the same orientation in CS and Svevo, and the total length of the region was similar in both species (5.2 Mb), suggesting that no major structural rearrangements occurred in the candidate gene region.

Finally, we did a BLAST comparison of all the Svevo genes to a Kronos scaffold assembly from the Earlham Institute, UK, and were able to detect 27 of the 28 genes with 100% identity. The only exception was *TRITD3Av1G056250* (ortholog of CS *TraesCS3A02G142600*), for which we only detected the B-genome homeolog in Kronos. These results suggest the Kronos genome is not very different from Svevo in this region. We currently do not know the cause of the reduced recombination frequency between 121.5 and 125.1 Mb in the KxG population, but since no pseudomolecule assembly of Kronos or Gredho is available, we cannot rule out the possibility of structural rearrangements in this region in one of these two varieties.

### Effect of *FT-A2* D10A polymorphism on heading time and fertility

Wheat varieties are selected to flower within a narrow time window to maximize grain productivity. This limits the introgression of loss-of-function alleles that have beneficial effects on SNS but generate large delays in heading time, such as those in *VRN1* (Li et al. 2019) or *PPD1* (Shaw et al. 2013)*.* By contrast, the *FT-A2* A10 allele has a positive effect on SNS and limited effect on heading time. Even when loss-of-function mutations in *ft-A2* and *ft-B2* were combined in Kronos, the delay in heading time was only 2–4 days (Shaw et al. 2019). In this study, the D10A polymorphism showed small effects on DTH in different genetic backgrounds, ranging from a non-significant difference in the initial Kronos x Gredho population (Table 2), a marginally non-significant difference of 0.8 d (*P* = 0.053) in the 2021 field experiment comparing Kronos isogenic lines, and an average difference of 1.7 d in the winter wheat population (Fig. 1A).

An important limitation for the utilization of the *ft-A2* loss-of-function mutation for wheat improvement was its negative effect on fertility (Shaw et al. 2019), which offset its positive effect on SNS, as confirmed in the 2021 Tulelake experiment in this study (Table 3). This motivated our initial search for *FT-A2* natural variants that separated the positive effects on SNS from the negative effects on fertility. Results presented in this study show that the positive effect of the A10 polymorphism on SNS were translated into positive effects on GNS in both the winter wheat population (Fig. 1e) and in the spring NILs (Table 3). In addition, this allele was not associated with negative effects on the number of grains per spikelet in any of the studied populations, suggesting that the A10 allele has no negative effect on fertility. These results provide a good example of the value of using natural variants selected by breeders to identify mutations that optimize specific traits with limited negative pleiotropic effects.

### *FT-A2* effects on SNS, GNS, grain weight and spike yield

It was encouraging to see that the positive effect of the A10 allele on SNS and GNS was expressed in both winter (Fig. 1) and spring wheats (Table 3), and among the latter in both spring and fall planted spring wheats. However, the magnitude of the increases in SNS, GNS, and spike yield associated with the A10 allele varied among experiments, suggesting that the effects of this *FT-A2* polymorphisms on these traits are modulated by the environment. We also observed variable effects of the A10 polymorphisms on grain weight. Whereas no significant effects were detected for this trait in the experiments performed at UCD in 2020 and at Tulelake in 2020 and 2021, we detected a significant reduction in grain weight in the field experiment performed at UCD in 2021, which offset the gains in GNS (Table 3).

Similar observations have been reported for *WAPO-A1*, the causal gene of a wheat SNS QTL on the long arm of chromosome 7AL (Kuzay et al. 2019). Increases in SNS associated with the favorable *Wapo-A1b* allele were translated into significant increases in grain yield only when the favorable *WAPO-A1* allele was present in high-yielding/high-biomass genetic backgrounds and the plants were grown in a favorable environment. When the *Wapo-A1b* allele was present in poorly adapted varieties or when the lines were grown under water-limiting conditions, the plants did not have enough resources to fill the extra grains, resulting in a negative correlation between grain number and grain weight that limited the gains in grain yield (Kuzay et al. 2019). A study with elite CIMYT lines also highlighted the importance of genetic-by-environment interactions on the trade-offs between grain number and grain weight (Quintero et al. 2018). We hypothesize that environmental differences between our 2020 and 2021 field trials may have contributed to the observed differences in grain weight, in spite of the positive effects of the A10 allele on SNS and GNS detected in both years (Table 3). We also hypothesize that the introgression of the *FT-A2* A10 allele into more productive durum wheat varieties than Kronos may result in significant increases in total grain yield.

### *FT-A2* as a candidate gene for previously published SNS QTLs on chromosome arm 3AS

A QTL for DTH (*Qncb.HD-3A*) was previously mapped on chromosome 3A within a 400 Mb interval including *FT-A2* (DeWitt et al. 2021) in the LA95135 x SS-MVP47 population. We found in this study that LA95135 carries the D10 allele and SS-MVP47 the A10 allele, and after genotyping the population with the *FT-A2* marker, we found that the A10 allele was associated not only with a slight delay in heading time but also with higher SNS, GN, and grain yield per spike (Fig. 1). The similar pleiotropic effects of the SNS QTL in the winter wheat population and the KxG population, together with the overlapping mapping regions, suggest that the *FT-A2* D10A polymorphism may have contributed to the *Qncb.HD-3A* identified in the LA95135 × SS-MVP47 population.

An additional QTL for DTH was identified in the Avalon x Cadenza population (U.K.) on chromosome arm 3AS around the peak marker BS00021976 (169 Mb RefSeq v1.0) (Martinez et al. 2021). This QTL interval (60 Mb at each side of BS00021976) includes 536 annotated genes, among which the authors proposed *FT-A2* as a candidate of particular interest. Using our *FT-A2* marker, we established that both Avalon and Cadenza carry the A10 allele, so we conclude that the D10A polymorphism is not the cause for the observed QTL for DTH on 3AS in this population. Martinez et al. (2021) suggested that differences in *FT-A2* transcript levels may contribute to the differences in DTH, but more precise mapping of this QTL will be necessary to support this hypothesis.

Several QTLs for grain yield components have been reported in different regions of chromosome 3AS in a recombinant inbred chromosome line from the cross between cultivar Cheyenne and a substitution of chromosome 3A of Wichita in Cheyenne (CNN(Wichita-3A)) (Ali et al. 2011; Campbell et al. 2003; Dilbirligi et al. 2006). QTLs for grain yield and grain number per square meter were mapped in a region between markers *barc86* and *barc67* (54.4 to 464.3 Mb RefSeq v1.0, “Region 2”) which encompasses the *FT-A2* locus. However, both Cheyenne and CNN(Wichita-3A) have the A10 allele of *FT-A2* (Supplementary Appendix S1), suggesting that a different gene (or a different polymorphism in *FT-A2*) was the cause of this QTL.

### *FT-A2* allele frequencies and breeding applications

The *FT-A2* alleles show contrasting frequencies in durum and common wheat, with the A10 allele present in less than 1% of the durum accessions and in 56% of the common wheat varieties analyzed in this study (Table 1). We currently do not know if the A10 allele originated in the few durum accessions carrying this allele in Oman and Turkey, or if these represent later introgressions from hexaploid to tetraploid wheat. Independently of its origin, the frequency of the A10 allele increased rapidly since its introgression or origin in common wheat, suggesting that this allele was favored by common wheat breeders.

The low frequency of the A10 allele in durum wheat could be a result of infrequent gene flow from hexaploid wheat to tetraploid wheat. However, it can also be the result of selection for larger grains and indirect selection for reduced GNS in environments showing a negative correlation between these two traits. Similar to *FT-A2,* the *Wapo-A1a* allele for low SNS is almost fixed in durum wheat, whereas the *Wapo-A1b* allele for high-SNS is found at high frequencies in hexaploid wheat (Kuzay et al. 2019). We interpret this similar asymmetric distribution of *WAPO-A1* and *FT-A2* alleles for SNS in common and durum wheat as indirect support for the hypothesis that selection for larger grains may have resulted in indirect selection for reduced SNS in durum wheat.

Among hexaploid spring wheats, we also observed significant differences in the distribution of the *FT-A2* alleles, with a larger frequency of the A10 allele among spring varieties developed under a long growing cycle (DuF, 58.4%) than among those developed under a short growing cycle (DuS, 34.4%). We speculate that longer cycles may provide more resources to fill the extra grains associated with the A10 allele, facilitating the translation of the difference in SNS into differences in grain yield. This in turn may result in a positive selection pressure for the A10 allele in the fall-planted programs. This idea is indirectly supported by the high frequency of the A10 allele among the US winter wheat varieties (Table 1, 81.7%). Additional experiments with D10 and A10 NILs in different genetic backgrounds tested in different spring-planted and fall-planted locations will be necessary to test this hypothesis.

The high frequency of the A10 allele in the winter wheats and fall-planted spring wheats provides additional evidence that this allele has positive effects in the regions where these cultivars are grown. However, as the frequency of the A10 allele increases, the number of varieties that can benefit from its introgression decreases. By contrast, the A10 allele is almost absent from modern durum wheat breeding programs, providing an opportunity to benefit a large proportion of these varieties. To facilitate the testing and introgression of the A10 allele into durum wheat breeding programs, we deposited the Kronos NIL with the A10 allele in the NSGC (PI 699107). Kronos is a modern durum wheat variety with excellent pasta quality, which makes it a better donor parent than Gredho.

Our preliminary results suggest that the A10 allele may be more beneficial in fall planted than in the spring planted durum wheat programs, but additional experiments are necessary to test this hypothesis. It will be also interesting to investigate the combined effect of the A10 allele with alleles from other genes that also result in increases in SNS such as *Wapo-A1b* (Kuzay et al. 2019) and the *Elf3* allele from *T. monococcum* (Alvarez et al. 2016).

In summary, the genetic information provided in this study, together with the previous mutant information, provides strong evidence that *FT-A2* is the causal gene for the differences in SNS, GNS, and spike yield associated with this region on chromosome arm 3AS. The identification of the likely causal polymorphism (D10A) and the development of a perfect marker for this polymorphism in this study can accelerate the deployment of this favorable allele in wheat breeding programs worldwide.

## Supplementary Information

Below is the link to the electronic supplementary material.

Supplementary file1 (XLSX 62 kb)

Supplementary file2 (DOCX 962 kb)

## Data Availability

All data and materials described in this paper are available from the corresponding author upon request. The *FT-A2* introgression in Kronos is deposited in the National Small Grains Collection (PI 699107). PI accession numbers are provided for all germplasm used when available. The datasets retrieved and analyzed during the current study are available in the T3/Wheat exome capture database (https://wheat.triticeaetoolbox.org/).
