# Supplementary material for: Identification and characterization of a natural polymorphism in FT-A2 associated with increased number of grains per spike in wheat
Source: Theor Appl Genet. 2021 Nov 26;135(2):679–92. doi: 10.1007/s00122-021-03992-y (PMC8866389; doi:10.1007/s00122-021-03992-y)

**Supplementary Tables and Figures**

**Table S1** Primers for the markers used in this study

| **Marker** | **Position** |  | **Primers** |  | **Haplotype** | |
| --- | --- | --- | --- | --- | --- | --- |
| **KASP** ^a^ |  | **FAM** | **VIC** | **COM** | **A10** | **D10** |
| 3A-117.82 | 117,828,272 | CTCGTCGCCGTCCACCT**T** | CTCGTCGCCGTCCACCT**C** | CTCCCTTGGACATTCCCTCC | FAM | VIC |
| 3A-118.6-R1 | 118,623,070 | CTCCTCCTCAGGCCACG**G** | CTCCTCCTCAGGCCACG**A** | CAGGTGAGACAACGAGCAGG | FAM | VIC |
| 3A-120.2 | 120,227,651 | GTGACCTACGACGTTGGCA**T** | GTGACCTACGACGTTGGCA**G** | AGTTTTCTCTGGTCCGCTCG | FAM | VIC |
| 3A-121.4 | 121,482,459 | CTACCTCCGCCACTGTGA**G** | CTACCTCCGCCACTGTGA**A** | GGACCTTCACTGTAGCCGAC | FAM | VIC |
| 3A-122.540 | 122,540,617 | GTCGGATCTGTCGAGTTCTC**C** | GTCGGATCTGTCGAGTTCTC**T** | TGACAATGACGACGTGGACTA | VIC | FAM |
| 3A-125.4-R1 | 125,402,254 | TCATGGACCTTGTCGATGCT**A** | TCATGGACCTTGTCGATGCT**G** | CCCAGCACCGACCTCACA | VIC | FAM |
| 3A-126.5-L4 | 126,567,437 | GTCTAGCCGTCGCCGAT**C** | GTCTAGCCGTCGCCGAT**G** | TGCACCCATCTCAAATTTCCG | VIC | FAM |
| 3A-127.8 | 127,821,835 | TAAGGAAGAAGAAGGCCCC**A** | TAAGGAAGAAGAAGGCCCC**G** | AAGACGCTCATCGCCCGT | FAM | VIC |
| **CAPS** |  | **Forward** | **Reverse** | **Restriction enzyme** | **Band** | **Band** |
| 3A-121.64 | 121,646,195 | GGGTTCTTTGATACTGGCAATGT | AGCTTCGGGGTACTGCTGT | *BssH*II ^b^ | Low | High |
| FT-A2-L4 | 122,542,102 | GAGTCCTGTTTCCGTCTTCCCT | GTGTTGCCCATCTCGAATAT | *Sac*II ^c^ | Low | High |
| FT-A2-D10A | 124,172,909 | CCGGACAGAGCAATGGACTT | TGAGTAAGCAGTCTAGGAGCAT | *Apa*I ^d^ | Low | High |
| FTA2-R1 | 125,094,949 | TCGTCGTCGTCGTCATCATC | AGTGGATTCGTTTCACGCCT | *Hinf*l ^e^ | Low | High |

^a^ 94 °C for 15 min, 10 cycles of: 94 °C 20 s, -0.8 °C touch down from 65 to 57 °C 1 min, and 30 cycles of: 94 °C 20s, 57 °C 1 min.

^b^ 94 °C for 5 min, 35 cycles of: 94 °C 30 s, 55 °C 30 s, 72 °C 30 s, and a final cycle of: 72 °C for 5 min.

^c^ 94 °C for 5 min, 35 cycles of: 94 °C 30 s, 55 °C 30 s, 72 °C 40 s, and a final cycle of: 72 °C for 5 min.

^d^ 94 °C for 5 min, 10 cycles of: 94 °C 30 s, -0.8 °C touch down from 65 to 57 °C 20 s, 72 °C 30 s, followed by 30 cycles of: 94 °C 30 s, 57°C for 20 s, 72 °C for 30 s, and a final cycle of: 72 °C for 5 min.

^e^ 94 °C for 5 min, 35 cycles of: 94 °C for 30 s, 55 °C 20s, 72 °C 30 s, and a final cycle of: 72 °C for 5 min.

**Table S2** Evaluation of BC_1_F_3:5_ homozygous sister lines from recombinant line H2-14-18-5 in a field experiment performed at UC Davis in 2021. H2-14-18-5-#3-3 was homozygous for the recombinant chromosome and H2-14-18-5-#3-3 for the non-recombinant chromosome, and both carried the *FT-A2* A10 (G) allele. Control lines H2-14-#16-2 and K, H2-14-#1-4 are separate lines from family H2-14 homozygous for the *FT-A2* D10 (K) or the *FT-A2* A10 (G) alleles, respectively.

| Marker | Chr. 3AS CS | H2-14-18-5 | H2-14-18-5 | H2-14 | H2-14 |
| --- | --- | --- | --- | --- | --- |
|  |  | #3-3 | #3-5 | #16-2 | #1-4 |
| 3A-117.82 | 117,828,272 | K | G | K | G |
| 3A-120.23 | 120,227,651 | K | G | K | G |
| 3A-121.48 | 121,482,459 | G | G | K | G |
| FT-A2 | 124,172,909 | G | G | K | G |
| **SNS PHENO**. |  | G | G | K | G |
| 3A-127.8 | 127,821,835 | G | G | K | G |
| Number of plants | | 30 | 48 | 49 | 38 |
| SNS Avg | | 22.69 | 23.23 | 21.72 | 23.19 |
| *P* values D10 (K) vs A10 (G) | | 0.8056 | | < 0.0001 | |

**Supplementary Figures**

**Fig. S1** Expression levels of *FT2* homeologs in hexaploid (Chinese Spring) and tetraploid wheat (Kronos) **a** Transcript levels of *FT-A2*, *FT-B2* and *FT-D2* in leaves, spikes and stems and three developmental stages (Choulet et al. 2014). Developmental stages are based on Zadok’s scale z10= seedling, z23= three tillers, z30= spike 1 cm, z32= two nodes, z39= meiosis, z65= anthesis, z71= two days after anthesis (Zadok et al. 1974). **b** Quant-Seq data from developing spikes of Kronos spring line *vrn1 vrn2* (Li et al. 2020). **c** Unpublished RNAseq from our lab at the same spike development stages in Kronos wild type (*Vrn-A1*). VEG = vegetative stage, DR = double ridge, PDR = post-double ridge, and TS = terminal spikelet stage. TPM= transcripts per million. Values are transcripts per million based on four biological replications per stage in each experiments and error bars are s.e.m.


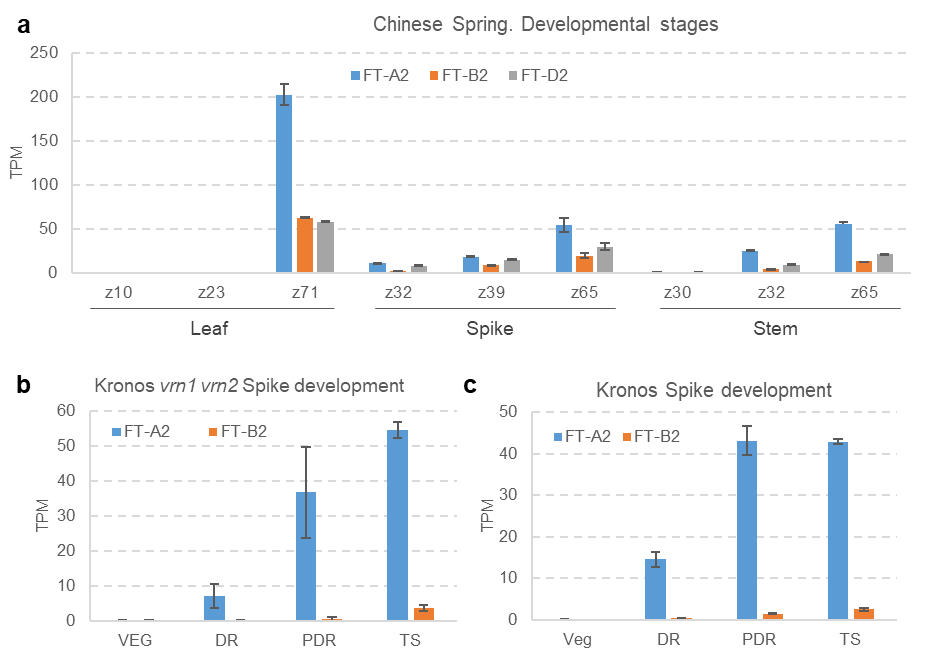


**References Fig. S1**

Choulet F et al. 2014. Structural and functional partitioning of bread wheat chromosome 3B. Science 345:1249721

Zadoks JC, Chang TT, Konzak CF. 1974.A decimal code for the growth stages of cereals. Weed Res. 14: 415-21.

Li K, Debernardi JM, Li C, Lin H, Zhang C, Dubcovsky J. 2020 Interactions between SQUAMOSA and SVP MADS-box proteins regulate meristem transitions during wheat spike development. bioRxiv:2020.2012.2001.405779

**Fig. S2** Yeast-two-hybrid interactions between FT-A2 and six 14-3-3 proteins. FT-A2 alleles D10 and A10 were used as baits and the six different 14-3-3 proteins as preys. Selection for yeast transformants containing both bait and prey vectors was performed in SD medium lacking Leucine and Tryptophan (-L-W). Interaction strength was tested on SD media lacking Leucine, Tryptophan, Histidine and Adenine (-L-W-H-A). As positive control, we used FT1 as bait and 14-3-3A as pray. As negative controls we tested the FT-A2 alleles D10 and A10 against the empty vector LAW11.


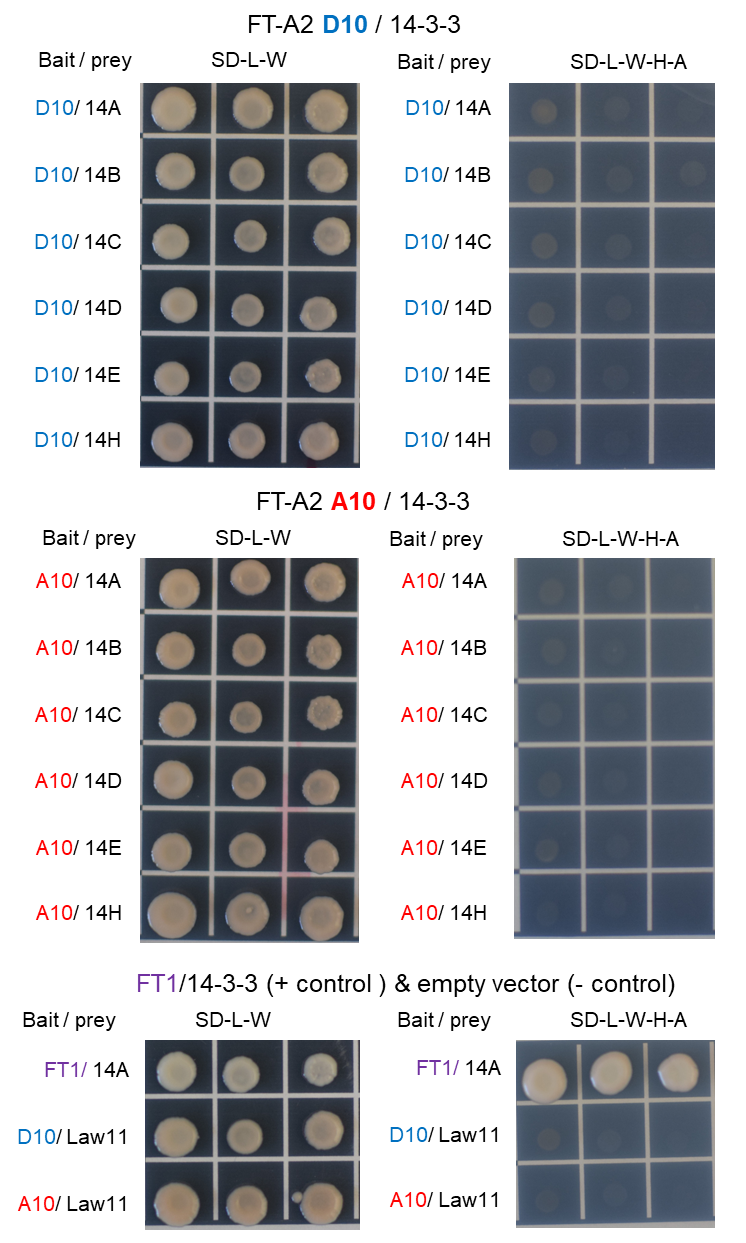

Supplement: Supplementary file 2 — Supplementary file2 (DOCX 962 kb) [file 122_2021_3992_MOESM2_ESM.docx]
